# Immunization with Hypoallergens of Shrimp Allergen Tropomyosin Inhibits Shrimp Tropomyosin Specific IgE Reactivity

**DOI:** 10.1371/journal.pone.0111649

**Published:** 2014-11-03

**Authors:** Christine Y. Y. Wai, Nicki Y. H. Leung, Marco H. K. Ho, Laurel J. Gershwin, Shang An Shu, Patrick S. C. Leung, Ka Hou Chu

**Affiliations:** 1 School of Life Sciences, The Chinese University of Hong Kong, Shatin, Hong Kong SAR, China; 2 Department of Pediatrics and Adolescent Medicine, The University of Hong Kong, Hong Kong SAR, China; 3 Department of Pathology, Microbiology and Immunology, School of Veterinary Medicine, University of California Davis, Davis, California, United States of America; 4 Division of Rheumatology/Allergy, School of Medicine, University of California Davis, Davis, California, United States of America; Centre de Recherche Public de la Santé (CRP-Santé), Luxembourg

## Abstract

Designer proteins deprived of its IgE-binding reactivity are being sought as a regimen for allergen-specific immunotherapy. Although shrimp tropomyosin (Met e 1) has long been identified as the major shellfish allergen, no immunotherapy is currently available. In this study, we aim at identifying the Met e 1 IgE epitopes for construction of hypoallergens and to determine the IgE inhibitory capacity of the hypoallergens. IgE-binding epitopes were defined by three online computational models, ELISA and dot-blot using sera from shrimp allergy patients. Based on the epitope data, two hypoallergenic derivatives were constructed by site-directed mutagenesis (MEM49) and epitope deletion (MED171). Nine regions on Met e 1 were defined as the major IgE-binding epitopes. Both hypoallergens MEM49 and MED171 showed marked reduction in their *in vitro* reactivity towards IgE from shrimp allergy patients and Met e 1-sensitized mice, as well as considerable decrease in induction of mast cell degranulation as demonstrated in passive cutaneous anaphylaxis assay. Both hypoallergens were able to induce Met e 1-recognizing IgG antibodies in mice, specifically IgG_2a_ antibodies, that strongly inhibited IgE from shrimp allergy subjects and Met e 1-sensitized mice from binding to Met e 1. These results indicate that the two designer hypoallergenic molecules MEM49 and MED171 exhibit desirable preclinical characteristics, including marked reduction in IgE reactivity and allergenicity, as well as ability to induce blocking IgG antibodies. This approach therefore offers promises for development of immunotherapeutic regimen for shrimp tropomyosin allergy.

## Introduction

Food allergy is a type 1 hypersensitivity disorder that affects up to 10% of the general population [1] and frequently lead to anaphylaxis. Food-related acute allergic reactions account for up to 49% of all anaphylaxis-related emergency department (ED) visits [2]–[4] and for patients discharged from ED, 54% filled epinephrine autoinjection prescription within one year [5]. Among all food allergies, shellfish allergy is one of the most common types with a prevalence of 0.6% in the world population [6], and is particularly common in Asian countries [7]. Shellfish is also considered as one of the four most common food, which could provoke anaphylaxis [8]. With an emerging trend in both shellfish production and consumption, the increase in the prevalence of shellfish allergy is predictable [9]. Improved clinical management of this disorder is therefore needed, and comprehensive studies of the molecular characteristics of shellfish allergens and therapeutic regimens are eminent.

At the molecular level, the muscle protein tropomyosin was identified as the major shrimp ingestion-related allergen in *Metapenaeus* and *Penaeus* spp [10]–[12]. Biochemically, tropomyosin is a coiled-coiled secondary structure protein of 34–38 kDa and functions in contractile activities of muscle cells [13]. While shrimp allergy has long been a model for studying shellfish allergy, our laboratory has cloned and expressed tropomyosin from *Metapenaeus ensis* (Met e 1), which exhibits specific serological IgE reactivity with serum samples from shrimp allergy patients [11]. This study has facilitated the subsequent identification of tropomyosin as an allergen common in crustaceans and mollusks [14]–[18]. Greatly attributed to the high amino acid sequence homology among the crustaceans and mollusks tropomyosins (93.8% and 77.2%, respectively), as well as a 61.4% sequence homology between the arthropods and mollusks tropomyosins, this protein is believed to be the major cross-reactive shellfish pan-allergen [13], [19]. Specifically, there are more than 99% sequence homology between the two most common reference shrimp allergens Met e 1 and the tropomyosin from *Penaeus aztecus* (Pen a 1) [12]. Met e 1 and Pen a 1 are therefore ideal model allergens, to be engineered for shrimp allergy immunotherapy studies but also possibly at other tropomyosin-induced shellfish allergies.

Although food avoidance and epinephrine injection are currently the first-line treatments in patients with anaphylaxis, allergen-specific immunotherapy (SIT) is the major strategy for clinical management of allergy as it has the capacity to modify the course of the disease. However, conventional modalities for SIT using native allergens are constrained due to the potential risk of allergic side-effects during treatment. In this context, hypoallergen with low/no IgE reactivity is desirable for SIT. Notably, the nature of allergenic epitopes and hypoallergens might greatly affect the SIT outcome such as the induction and generation of blocking antibodies, shifting of the Th1/Th2 paradigm and induction of peripheral tolerance by recruitment of regulatory T cells [20]–[25]. Molecular characterization of allergens, exemplified by the identification of IgE-binding epitopes, is thus imperative for the design of safer immunotherapy regimens [26]. Ayuso et al. have applied the concept of a hypoallergenic mutant by introducing 12 point mutations into the eight IgE-binding epitopes [27] within the five allergenic regions of Pen a 1 [28]. Although this mutant showed a reduction of allergenic potency of 90–98% in humanized rat basophilic leukemia (RBL) release assay, maximal releases were similar between the mutant and wild-type Pen a 1. This result suggests that other significant allergenic epitopes may exist in addition to the eight allergenic sites reported, thus additional approaches are necessary to construct a hypoallergen of shellfish tropomyosin.

To circumvent this issue, we have chosen a two-pronged approach in designing shrimp tropomyosin hypoallergens. In this study, the first objective is to define the major IgE-binding epitopes of *Metapenaeus* tropomyosin Met e 1. The second objective of this study is to construct hypoallergenic derivatives of Met e 1 by introducing point mutations within the IgE-binding epitopes identified, or by deleting these epitopes. The IgE reactivity, allergenicity, immunogenicity and the inhibitory potential of the hypoallergen-induced antibodies towards IgE antibodies of subjects allergic to shrimp and Met e 1-sensitized mice [29] are characterized and compared to the wild type allergen Met e 1. Herein, we specifically used serum samples from children and adolescents allergic to shrimp in mapping the IgE-binding epitopes. Previous study reported greater epitope diversity among children allergic to shrimp than adult patients [30] and outgrown of shellfish allergy is rarely reported [31], [32]. We therefore believe that the use of pediatric serum samples could resolve an epitope profile of Met e 1 that is comprehensive, clinically relevant and common among shrimp allergy patients in any age group. The hypoallergens constructed based on this epitope profile should also be applicable in immunotherapy targeting at both pediatric and adult patients.

## Materials and Methods

### Serum samples

Serum samples were obtained from 12 subjects (aged 3–17 years) with confirmed clinical history of allergic responses to shrimp and positive skin prick test (Table S1). Specific IgE reactivities to purified recombinant shrimp tropomyosin Pen a 1 and Met e 1 were characterized by ImmunoCAP and ELISA, respectively. None of the recruited subjects have other allergies. Serum samples (n = 8) obtained from healthy, non-atopic volunteers without Met e 1-specific IgE were used as a negative control.

### Ethics statement

A written consent was obtained from the parents of the children enrolled in the study (Institutional Review Board of the University of Hong Kong/Hospital Authority Hong Kong West Cluster, Ref. No. UW10-115). The use and storage of human sera were approved by the Joint Chinese University of Hong Kong - New Territories East Cluster Clinical Research Ethics Committee with a written informed consent (CREC Ref. No. CRE-2010-514). All animal protocols were approved by the Animal Experimentation Ethics Committee, The Chinese University of Hong Kong (ref No. 11/006/GRF and 463911), in accordance with the Department of Health (Hong Kong) guidelines in Care and Use of Animals. All experiments were performed under licenses granted from the Government of Hong Kong Special Administrative Region.

### Identification of allergenic epitopes

There were three independent methods used to predict the immunodominant allergenic epitopes including 1) computational prediction of IgE binding epitopes, 2) ELISA against overlapping peptides that span the entire Met e 1 sequence, and 3) dot-immunoblotting of overlapping peptides against the entire Met e 1 sequence. 18 overlapping peptides spanning the full-length (274 amino-acids) Met e 1 were commercially synthesized (GenScript). Each peptide had 20 amino acids (except for peptide 18 that contains 19 amino acids) with five amino acids overlapping with the adjacent peptides at the N-terminus. Individual peptides were dissolved in distilled water, aliquoted and stored at −20°C until required.

1. Three computational models from the Immune Epitope Database (IEDB) Analysis Resource were employed to predict the major linear IgE-binding epitopes of Met e 1, including Bepipred Antibody Epitope Prediction, Kolaskar & Tongaonkar Antigenicity model and Emini Surface Accessibility Prediction. Bepipred Antibody Epitope Prediction predicts the location of IgE-binding epitopes based on the hidden Markov model and propensity scale method [33]. The Kolaskar & Tongaonkar Antigenicity model is based on the physiochemical properties of amino acid residues [34]. Emini Surface Accessibility Prediction is based on the calculation of the surface accessibility scale [35].
2. For peptide ELISA, 3 µg of each peptide were coated on 96-well plates (Nunc, maxisorp) in 0.05 M carbonate buffer overnight. After blocking with 1% BSA/PBS for 1.5 h, the plates were incubated with individual serum samples (150 dilution) at room temperature for 2 h. Thereafter, the plates were incubated with biotinylated goat anti-human IgE (Vector) in 11000 dilution for 45 min followed by incubation with Avidin D, Peroxidase labeled antibody (Vector) in 11000 dilution for 30 min. The plates were then developed with TMB substrate reagent set (BD Biosciences) for 15 min and the reaction was terminated by 2 N H_2_SO_4_. Absorbance was measured at 450 nm using an ELISA plate reader (Bio-Rad). All absorbance values were background-corrected, in which the background absorbance was the OD value of Met e 1-coated wells incubated with secondary and tertiary antibodies only. All the above procedures were performed at room temperature. The plates were washed with PBS/0.5% Tween-20 (PBST) three times and PBS once between each step and all dilutions were made in 1% BSA/PBS.
3. For dot-immunoblotting, 3 µg of each peptide (3 µL) were spotted onto a 0.2 µm nitrocellulose membrane (Bio-Rad). The membrane was allowed to air-dry and thereafter fixed with 2.5% glutaraldehyde/PBS for 10 min [36]. The membrane was incubated with diluted serum (150 dilution) overnight at 4°C after a 2-h blocking in 3% skim milk/TBS. The membrane was incubated with mouse monoclonal anti-human IgE-alkaline phosphatase antibody (Sigma Aldrich) at 12000 dilution for 1 h at room temperature followed by signal development with NBT/BCIP (Roche). All dilutions were made with 3% skim milk/TBS and all washing steps were performed with TBST) once and TBS three times with shaking.

### Design of hypoallergenic shrimp tropomyosins

**Figure 1:**
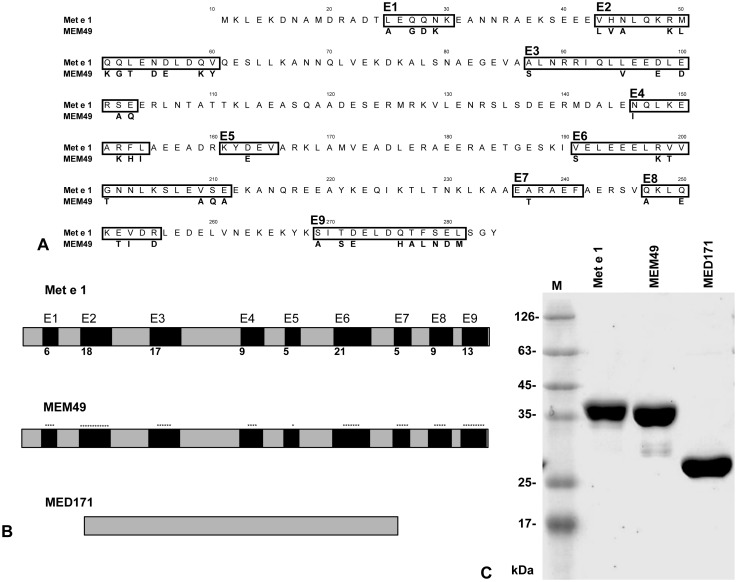
Design of two hypoallergenic mutants. MEM49 was constructed by substitution of 49 different amino acid residues within the nine Met e 1 epitopes to the homologous fish tropomyosin sequence. MED171 was constructed by deletion of epitopes E1 to E9 of Met e1. (**A**) Location of the IgE-binding epitopes in tropomyosin. The IgE epitopes designated as E1–E9 are shown in boxes and the location of the 49 amino acid residues in Met e 1 that are converted in MEM49 are also shown as one letter amino acid code. (**B**) Schematic representation of Met e 1, MEM49 and MED171. Epitopes E1 to E9 in Met e 1 are represented as black boxes and the number of amino acids in each epitope is indicated. Amino acid residue changes in MEM49 are shown as *. MED171 is a truncated peptide with the epitopes E1–E9 deleted. (**C**) SDS-PAGE of Met e 1, MEM49 and MED171 after Coomassie Blue staining. Note the 35 kDa molecular weight of Met e 1 and MEM49 and the expected smaller size of MED171 compared to Met e 1.

With the high structural flexibility and spontaneous unfolding property of tropomyosin [37], we believe that the possibility of having only one single amino acid per epitope that is crucial for IgE binding is unlikely. Restricted homologous substitution may not be sufficient to result in tropomyosin variants with greatly reduced IgE reactivity. Therefore, the amino acid sequence of Met e 1 was compared to the non-allergenic fish tropomyosins of four species *Salmo salar* (Atlantic salmon; GenBank accession number NP_001117128.1), *Epinephelus coioides* (orange-spotted grouper; ADG29138.1), *Siniperca chuatsi* (Mandarin fish; AEK21799.1) and *Thunnus thynnus* (Atlantic bluefin tuna; BAD01050.1) (Fig. S1). All nine identified IgE-binding regions in Met e 1 were converted into the homologous sequence of fish tropomyosins and 49 point mutations in total were introduced to construct the mutation mutant MEM49 (Fig. 1A & B). To construct the deletion mutant, all nine IgE-binding epitopes were deleted (Fig. 1B). This mutant, named MED171, contained only 171 amino acid residues. The amino acid sequences of MEM49 and MED171 were reverse translated using MEGA 5.0 and the encoding sequences of the two mutants were synthesized commercially by GenScript. Synthetic genes of each of the mutants were cloned into pET30(a)+ (Novagen) expression vector via *EcoRV* and *HindIII* restriction sites. The sequences and reading frame of MEM49 and MED171 in the plasmid were confirmed by dideoxynucleotide sequencing.

### Preparation of recombinant wild type and mutant shrimp tropomyosin

cDNA coding for the full length shrimp tropomyosin Met e 1 and the encoding sequences of MEM49 and MED171 were cloned into His-tag expression vector pET30(a)+ (Novagen) and expressed in *Escherichia coli* BL21 (DE3) (Invitrogen) by culturing in MagicMedia (Invitrogen) at 37°C overnight. His-tagged recombinant Met e 1 (rMet e 1), MEM49 and MED171 were purified using the HisPur Cobalt Spin Column (Thermo Scientific) according to the manufacturer’s instructions. Protein concentration was determined by BCA assay (Sigma Aldrich) while the purity was determined by Sodium dodecyl sulfate-polyacrylamide gel electrophoresis (SDS-PAGE) and Coomassie blue staining.

### Mice sensitization and immunization

3–4 weeks old female Balb/c mice were acquired from the Laboratory Animal Services Centre, The Chinese University of Hong Kong. All animals were maintained on a shrimp-free diet and housed in pathogen-free conditions. To induce Met e 1 hypersensitivity in mice, sensitization was performed as described previously by intragastric administration of 0.1 mg of recombinant tropomyosin plus cholera toxin on days 0, 12, 19 and 26 and challenged on day 33 [29]. Mice fed with phosphate-buffered saline plus cholera toxin were included as controls. Blood samples were collected 4 h after the challenge for antibody analysis.

For immunization experiments, 5–6 weeks old female Balb/c mice were intraperitoneally immunized three times on days 0, 7 and 14 with 0.1 mg purified rMet e 1, MEM49 or MED171 adsorbed to 1 mg Al(OH)_3_. Blood was collected 4 h after the last injection for the determination of antibody levels.

### Direct ELISA

To examine the IgE reactivity to rMet e 1, MEM49 or MED171, 96-well ELISA plates were coated with 5 µg/mL of either rMet e 1, MEM49 or MED171 in 0.05 M carbonate buffer overnight at 4°C, blocked with 1% BSA/PBS for 2 h and incubated with serum samples from shrimp allergy subjects or Met e 1-sensitized mice (110 dilution) overnight at 4°C. After washing, plates were incubated with biotinylated anti-human (Vector) or anti-mouse IgE antibodies (BD Pharmigen) and Avidin D, Peroxidase labeled antibody (Vector), each at 11000 dilution at room temperature for 1 h and 30 min, respectively. The plates were then developed with TMB substrate reagent set (BD Biosciences) for 15 min and the reaction was terminated by 2 N H_2_SO_4_.

To determine the reactivity of IgG and IgG_2a_ antibodies raised in rMet e 1, MEM49 and MED171 immunized mice, sera in serial dilutions (1400 to 125600) were incubated in the rMet e 1, MEM49 or MED171 coated plates (5 µg/mL) for 2 h at room temperature. The plates were then incubated with goat anti-mouse IgG or anti-mouse IgG_2a_ (Southern Biotech) in 12000 dilution for 45 min followed by incubation with Avidin D, Peroxidase labeled antibody (Vector) in 11000 dilution for 30 min. The plates were then developed with TMB substrate reagent set (BD Biosciences) for 5 min and the reaction was terminated by 2 N H_2_SO_4_.

### Passive cutaneous anaphylaxis

Passive cutaneous anaphylaxis was performed to determine the *in vivo* allergenicity of MEM49 and MED171. Backs of naïve Balb/c mice were shaved, followed by intradermal injection of Met e 1-specific IgE-containing sera (undiluted sera in a total volume of 100 µL) under isoflurane narcosis. Two hours later, mice were injected intravenously with a mixture of 100 µL of 0.5% Evan’s blue dye (Sigma Aldrich) and 0.1 mg rMet e 1, MEM49 or MED171. Thirty minutes after dye-rMet e 1 administration, mice were sacrificed by cervical dislocation and skins of their backs were immediately inverted for the measurement of blue region diameters.

### Competitive inhibition ELISA

Competitive inhibition ELISA was performed to evaluate the blocking capacity of hypoallergen-induced blocking antibodies. Briefly, rMet e 1 was used to coat 96-well plates (5 µg/mL) overnight at 4°C and blocked with 1% BSA/PBS for 2 h. Plates were then washed and blocked with 100 µL of 110 diluted sera from mice immunized MEM49, or MED171 overnight at 4°C. Thereafter, 100 µL of sera from shrimp allergy patients or Met e 1-sensitized mice at a predetermined dilution (110–120 dilution) were added and incubated at room temperature for 2 h. The wells were then washed and followed by the addition of biotinylated anti-human or anti-mouse IgE antibodies, HRP-Avidin D and developed as described above. The blocking ability of the induced IgG antibodies was determined using the equation [(OD_no inhibitor_–OD_inhibitor_)/OD_no inhibitor_]×100% and expressed as percentage inhibition.

### Statistical analysis

Data were presented as mean ± SEM. The statistical comparison was determined by one-way analysis of variance (ANOVA) followed by the Student-Newman-Keuls test using SigmaStat 3.1. The difference was considered statistically significant at p<0.05.

## Results

### IgE-binding epitopes of Met e 1 and hypoallergen design

**Figure 2:**
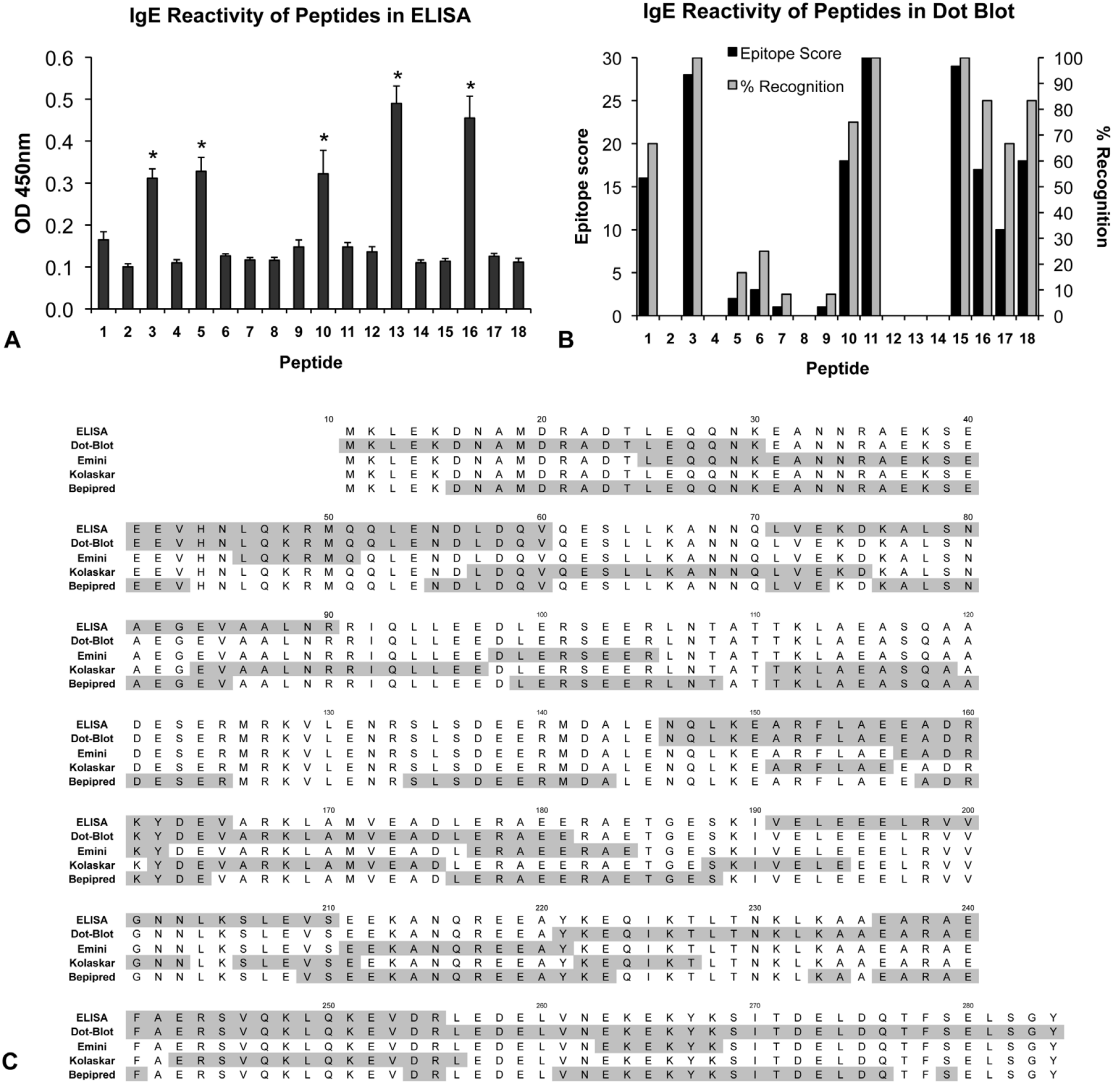
Determination of Met e 1 IgE-binding epitopes. Epitopes were determined by ELISA, dot-immunoblotting and three prediction models Emini Surface Accessibility Prediction Kolaskar & Tongaonkar Antigenicity model and Bepipred Antibody Epitope Prediction. (**A**) Histogram of the IgE binding reactivity against the Met e 1 peptides as determined by ELISA. (**B**) Histogram of IgE binding reactivity against the Met e1 peptides as determined by dot-immunoblotting. (**C**) Alignment of Met e 1 IgE-binding epitope sequences as determined by ELISA, dot-immunoblotting and each of the three prediction models.

By ELISA, sera from patients with shrimp allergy (n = 12) had significantly higher IgE reactivity against five peptides (P3, P5, P10, P13 and P16) when compared with other peptides (p<0.05) (Fig. 2A). None of the sera from control subjects (n = 8) showed IgE-binding activity towards these or other peptides (data not shown).

Allergenic regions on Met e 1 were also defined based on the intensity of peptide spots and the frequency of recognition in dot-immunoblotting (Fig. 2B). A peptide with >50% recognition (6 out of 12 patients) or an epitope score (calculated by the summation of the IgE reactivity score (strong reactivity: 3; median: 2; low: 1)) higher than the mean intensity score (8.83, calculated by adding all epitope scores and dividing by 18 peptides) was defined as a major IgE-binding epitope. Based on these criteria, eight peptides (P1, P3, P10, P11, P15, P16, P17 and P18) were identified as the major Met e 1-specific IgE-binding sequences. The discrepancy in epitopes determined by ELISA and dot-immunoblotting (Fig. 2C) was apparently due to assay sensitivity and peptide presentation on different materials in the two assays.

Three online immunoinformatics models were applied to define the IgE epitopes. (Fig. 2C & Fig. S2). Seven epitopes, with six to 16 amino acid residues in length, were identified using Emini Surface Accessibility Prediction based on the surface probability score (Fig. S2A). Ten allergenic regions, between six to 19 amino acid residues in length, were defined under the Kolaskar & Tongaonkar Antigenicity model based on the antigenic propensity score (Fig. S2B). Using Bepipred Antibody Epitope Prediction, 15 regions from one to 28 amino acid residues in length were recognized as IgE-binding epitopes (Fig. S2C). In comparing the predictions by these three models, Emini Surface Accessibility Prediction and Bepipred Antibody Epitope Prediction yielded very similar epitope results (>85% similarity, calculated as the degree of overlapping amino acid residues), while the prediction by Kolaskar & Tongaonkar Antigenicity deviated from those of the other two models. Only six regions resulted in consensus between Emini Surface Accessibility Prediction and Kolaskar & Tongaonkar Antigenicity, but with a low degree of overlap ranging between 14% and 37%.

Data obtained by ELISA and dot-immunoblotting, as well as from the three predictions models, were combined and equally weighted for defining the major IgE-binding epitopes (Fig. 2C). Logically, sequences that are determined as IgE reactive both experimentally and by modeling studies are more likely to represent IgE-binding epitopes in the native protein. Therefore, only regions that were suggested as IgE reactive by at least one of the experimental assays, and at least two other of the above assays or models, were considered as major epitopes [38]. Altogether, nine major IgE-binding epitopes of Met e 1 ranging from five to twenty-one amino acid residues in length were identified, namely E1–E9, with positions at Met e 1^25–30^, Met e 1^43–60^, Met e 1^87–103^, Met e 1^146–154^ Met e 1^161–165^, Met e 1^191–211^, Met e 1^236–241^, Met e 1^247–255^ and Met e 1^269–281^, respectively (Fig. 1A). Based on these epitopes, we constructed two tropomyosin mutants, by site-directed mutagenesis (MEM49) and epitope deletion (MED171). The locations of the IgE epitopes and their corresponding amino acid changes in mutants MEM49 and MED171 are shown in Fig. 1A and B. Approximately 4 mg of purified soluble recombinant proteins of MEM49 and MED171 could be obtained from 1 liter of *E.coli* culture. SDS-PAGE analysis of purified recombinant proteins of the mutation mutant MEM49 and the deletion mutant MED171 showed a 35-kDa MEM49 band and a 27-kDa MED171 band, compared to a 35 kDa rMet e 1 band (Fig. 1C).

### Immunoreactivity of tropomyosin mutants

**Figure 3:**
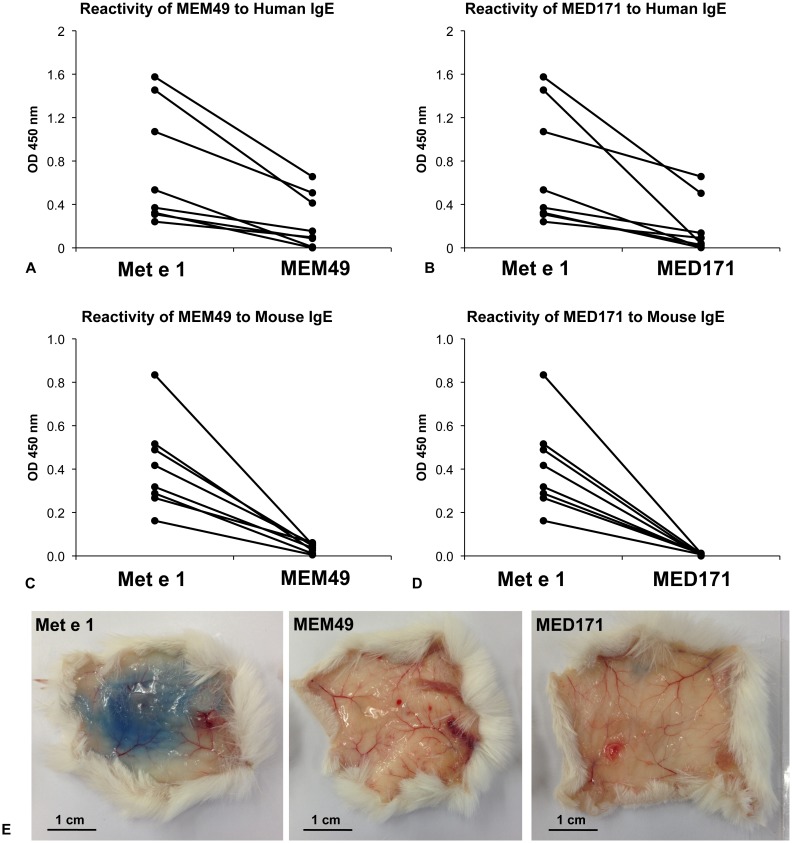
*In* *vitro* and *in* *vivo* IgE reactivity and allergenicity of the hypoallergens. Reactivity of (**A**) MEM49 and (**B**) MED171 to IgE from shrimp allergy patients (n = 8) and reactivity of (**C**) MEM49 and (**D**) MED171 to IgE of Met e 1-sensitized mice (n = 8) in ELISA. (**E**) *In vivo* IgE reactivity and allergenicity of Met e 1, MEM49 and MED171 as determined by PCA assay. Note that the *in vitro* and *in vivo* IgE reactivity and allergenicity of MEM49 and MED171 are significantly lower than those of Met e 1, as shown by the significantly lower absorbance value at 450 nm and absence of Evan’s blue dye extravasation.

Sera from 8/8 shrimp allergy patients and Met e 1-sensitized mice showed a marked decrease in IgE reactivity to MEM49 and MED171 (Fig. 3). Reactivity of MEM49 and MED171 towards patient IgE decreased by an average of 71.4% and 77.4% relative to Met e 1, respectively (Fig. 3A & B), while that to mouse IgE decreased by an average of 90.5% and 97.6%, respectively (Fig. 3C & D). Notably, the IgE-binding reactivity of MED171 was significantly lower than that of MEM49 (p<0.05) when tested with mouse sera. In addition to *in vitro* reduction in IgE reactivity, both MEM49 and MED171 did not trigger mast cell degranulation in passive cutaneous anaphylaxis assays. In contrast to a >2.5 cm blue region induced by intradermal injection of Met e 1-specific IgE and intravenous injection of Met e 1 with Evan’s blue dye, no Evan’s blue dye extravasation could be induced by intravenous injection of either hypoallergens (Fig. 3E). More importantly, none of the MEM49- or MED171-immunized mice produced Met e 1-recognizing IgE antibodies (OD 0.071±0.001 and 0.092±0.003, respectively) and hypoallergen-specific IgE antibodies, comparing to an IgE level of OD 0.405±0.056 upon Met e 1 immunization (Table 1). These clearly demonstrated that both MEM49 and MED171 had marked reduction in their *in vivo* allergenicity.

**Table 1:** Immunoreactivity of mutants as hypoallergen vaccines in mouse.

Group	Met e 1				MEM49				MED171			
	IgE		IgG		IgE		IgG		IgE		IgG	
	no. of micereacted	OD	no. of micereacted	OD	no. of micereacted	OD	no. of micereacted	OD	no. of micereacted	OD	no. of micereacted	OD
**Immunized** **with rMet e 1**	6	0.405±0.056	6	1.778±0.037	0	0.038±0.008	6	1.733±0.054	0	0.08±0.002	6	0.754±0.087
**Immunized** **with MEM49**	0	0.071±0.01	6	0.571±0.082	0	0.081±0.002	6	1.852±0.319	0	0.069±0.006	6	0.283±0.015
**Immunized** **with MED171**	0	0.092±0.003	6	1.089±0.085	0	0.069±0.009	6	0.857±0.073	0	0.089±0.005	6	1.121±0.098

Balb/c mice (n = 6 in each group) were immunized with rMet e 1, MEM49 or MED171 and their serological IgE and IgG reactivity were analyzed. Note that IgE is only induced in the rMet e 1-immunized mice and IgG to Met e 1, MEM49 and MED171 are cross-reactive.

### Hypoallergen-immunized mice produced Met e 1-specific IgG antibodies and inhibited IgE binding to Met e 1

**Figure 4:**
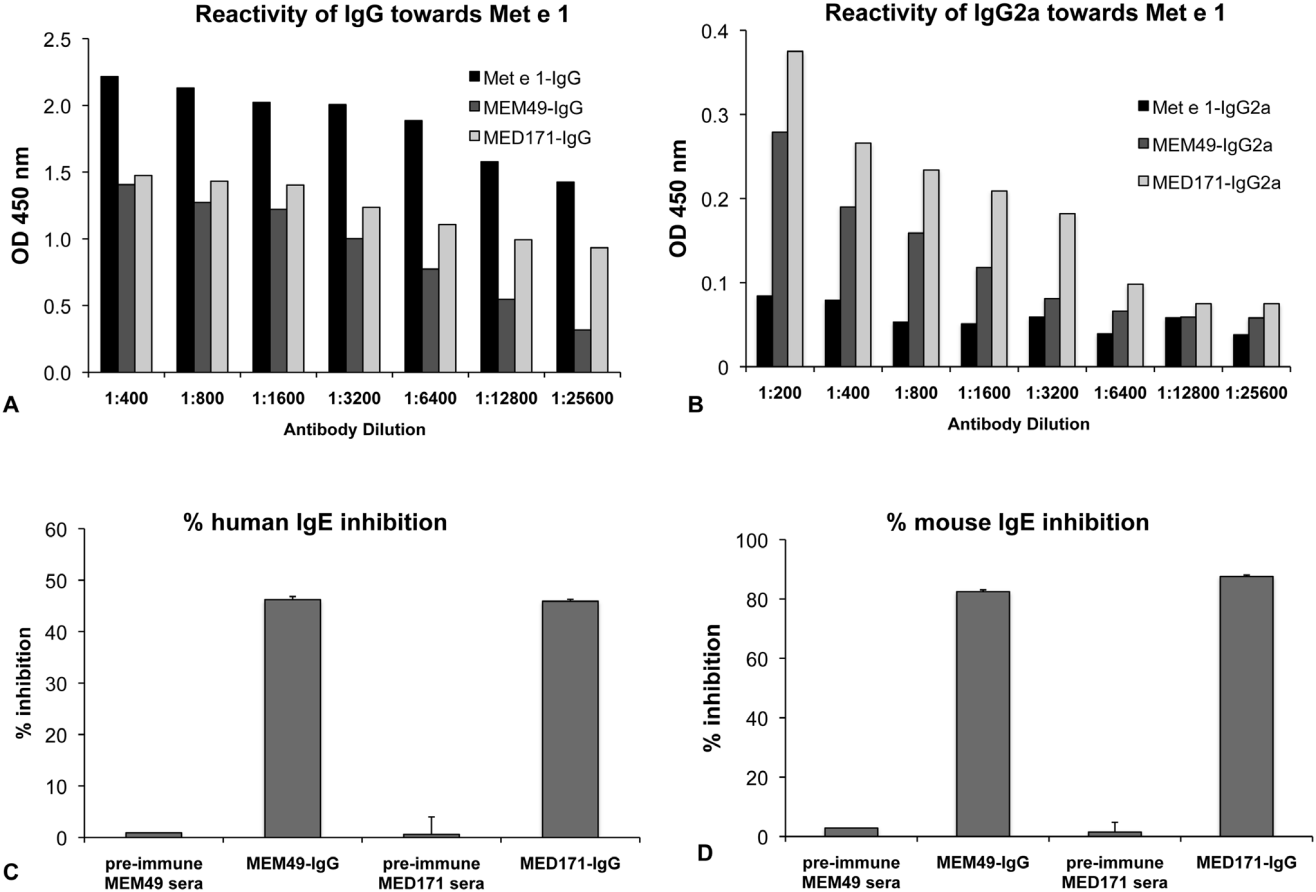
Immuno-reactivity of hypoallergens and inhibitory potential of the induced IgG antibodies. Reactivity of the rMet e 1-, MEM49- and MED171-induced (**A**) IgG and (**B**) IgG_2a_ antibodies towards the wild type allergen rMet e 1. Note that specific IgG_2a_ could only be induced by the hypoallergens. Inhibitory potential of the induced IgG towards Met e 1-specific IgE from (**C**) shrimp allergy subjects (n = 8) and (**D**) Met e 1-sensitized mice (n = 8) determined by competitive inhibition ELISA. Percentage inhibition was calculated by [(OD_no inhibitor_–OD_inhibitor_)/OD_no inhibitor_]×100%. Note that the MEM49- and MED171-induced IgG antibodies could significantly inhibit IgE of shrimp allergy patients and Met e 1-sensitized mice from binding to Met e 1.

Mice immunized with either rMet e 1, MEM49 or MED171 produced robust IgG antibodies that recognized rMet e 1 with OD 1.778±0.037, 0.571±0.082 and 1.089±0.085, respectively (Table 1). Moreover, IgG antibodies induced by MED171 exhibited better rMet e 1 recognition when compared to those induced by MEM49 at all tested dilutions (Fig. 4A). It is noteworthy that only the hypoallergens MEM49 and MED171, but not Met e 1, could induce the production of Met e 1-specific IgG_2a_ antibodies (Fig. 4B). We further examined if the sera IgG antibodies from hypoallergen-immunized mice were able to block Met e 1-specific IgE from binding to rMet e 1 by competitive inhibition ELISA. Serological IgG from MEM49 and MED171 were able to inhibit 46.2±3.41% and 45.9±3.54% of IgE from shrimp allergy patients from binding to Met e 1, respectively (Fig. 4C). MEM49- and MED171-IgG could better inhibit mouse IgE binding to Met e 1 with average of 82.5±3.24% and 87.6±2.84%, respectively (Fig. 4D).

## Discussion

Knowledge of the IgE-binding epitopes of allergens is fundamental for designing hypoallergenic derivatives, which are regarded as one of the best candidates applicable in SIT. Successful SIT using hypoallergens has been well demonstrated in mouse models of respiratory allergies [39]–[42] as well as in clinical trials on birch pollen allergy patients [43]–[45]. The fish parvalbumin mutant Mut-CD/EF that displays a 95% reduction in IgE reactivity and ability to induce blocking IgG antibodies might represent the only best-known hypoallergen among all the most common food allergens [46]. Meanwhile, hypoallergens of the major shellfish allergen tropomyosin that could be translated into specific immunotherapy are unavailable.

Although several shrimp allergens including arginine kinase [47], [48], sacroplasmic calcium-binding protein [49], [50], myosin light chain [51], [52] and troponin C [51] have been identified and registered by the IUIS-allergen database, tropomyosin is reactive to >80% patients allergic to shrimp and is regarded as the major shrimp and shellfish cross-reactive allergen [10], [11]. Herein, we have defined the IgE-binding epitopes of the shrimp tropomyosin Met e 1 by ELISA, dot-immunoblotting and three online models as prediction tool represents an emerging strategy in epitope mapping studies among food and drug allergies [38], [53], [54]. Using this combination, we aimed to achieve higher accuracy, including a lower chance of missing important epitopes, more complete recovery and a higher resolution of epitopes. Using this approach, nine major IgE-binding Met e 1 epitopes were identified. These epitopes range from five to twenty one amino acid residues in length, with some of these allergenic regions longer than the IgE-binding epitopes of other allergens [53], [55]–[57]. This variation may be due to the relatively simple coiled-coiled secondary structure of tropomyosin and/or the high flexibility of this molecule [37], possibly resulting in the higher proportion of surface-exposing IgE-binding sequences. The discovery that six IgE-binding epitopes identified in our work overlap with those previously reported for Pen a 1 [27], [28] is not surprising because the two proteins only have one amino acid difference at residue 69. The three Met e 1 IgE epitopes (E1, E5 and E7) newly identified in this study (Fig. 2A) may partly account for the limited success of a Pen a 1 hypoallergen in reducing allergenicity to shrimp tropomyosin [27]. Incidentally, serum samples from adults were used in the Pen a 1 study while serum samples from children and adolescents were used in determining the IgE-binding epitopes of Met e 1. The presumed greater epitope diversity in children with shrimp allergy than adults [30] may account for the additional epitopes revealed in the present study. Interestingly, some of the Met e 1 epitopes predicted by Bepipred Antibody Epitope Prediction are only one to five amino acid residues apart. Although this model was designed for continuous B cell epitope prediction, a recent study suggests that the results are similar to the predicted discontinuous B cell epitopes [58]. Hence, the epitopes predicted by this model may possibly represent the discontinuous epitopes of Met e 1, although more sophisticated experiments such as crystal structure resolution of allergen/IgE complex could be conducted to confirm the identity of the discontinuous epitopes of Met e 1. Nevertheless, the identification of previously unidentified IgE-binding epitopes in our study as compared to the study on Pen a 1 may be partly explained by the characterization of both linear and discontinuous IgE-binding epitopes here.

In the immunotherapy of allergy, a major goal is to reduce IgE-mediated side-effects during the course of immunotherapy. The two major strategies to reduce IgE reactivity include mutating the amino acid residues involved in IgE-binding, and disrupting the three-dimensional structure of the allergen [59]. Based on our IgE- epitope data, we constructed two hypoallergenic derivatives of Met e 1.

First, hypoallergen MEM49 was constructed by replacing 49 amino acid residues within the nine Met e 1 IgE-binding epitopes with the homologous tropomyosin sequences of fish. Tropomyosin sequences of more than ten fish species are available on GenBank. Herein, we have chosen tropomyosin sequences from four common edible fish species, *Salmo salar*, *Epinephelus coioides*, *Siniperca chuatsi* and *Thunnus thynnus* for comparison. To our knowledge, these fish tropomyosins have not been documented as ingestion-related allergens (however, see Liu et al. which shows that tilapia tropomyosin may be related to autoimmune diseases [60]) and are thus valid candidates for such a homologous conversion. The advantage of homologous substitution is that MEM49 would retain its natural conformation and thereby ensuring a strong allergen-specific IgG response [61]. On the other hand, we believe that with the high structural flexibility of tropomyosin and its spontaneous unfolding property [37], the possibility of having only one single critical amino acid per epitope that is responsible for IgE binding is unlikely. Therefore, restricted homologous substitution may not be sufficient to significantly reduce the IgE-binding reactivity of the variant. Hence, all the identified IgE-binding regions in Met e 1 were converted into the homologous sequence of fish tropomyosins.

The second hypoallergen MED171 was designed by deleting all IgE-binding epitopes, which results in a smaller-sized truncated tropomyosin variant of only 171 amino acid residues. With the disruption of all epitopes and possibly its structural flexibility as in tropomyosin, IgE reactivity and allergenicity of MED171 should be more significantly abolished. From our data, both variant showed significant reduction in their *in vitro* reactivity towards Met e 1-specific IgE from patients and sensitized mice. Both of them also lost their *in vivo* allergenicity in inducing mast cell degranulation or IgE synthesis. Direct ELISA also demonstrated that the IgE reactivity of MED171 is significantly lower than MEM49 when tested with sera from Met e 1-sensitized mice (2.4% IgE reactivity retained comparing to 9.5% in MEM49), which matches with our initial expectation.

We noted that most of the human shrimp tropomyosin CD4^+^ T cell epitopes mapped by Ravkov et al. [62] remain intact in both hypoallergens and therefore, both MEM49 and MED171 should retain their immunogenicity in inducing IgG antibodies. This is supported by our data that a robust Met e 1-specific IgG response was induced by MEM49 and MED171. Notably, we specifically detected the production of IgG_2a_ antibodies in mice immunized with MEM49 or MED171, but not with the wild type allergen Met e 1. The Th1-driven allergen-specific IgG_2a_ antibody in mouse and IgG_4_ antibody in human induced during SIT are considered to be blocking antibodies and correlate well with clinical improvements [63]–[71]. The fast-acting blocking IgG antibodies provides protection possibly through the formation of IgG/FcγRIIb complex on mast cells that down-regulates IgE receptor FcεRI signaling and mast cell degranulation [70], [72], sequestration of the circulating allergen by the induced IgGs [73], and/or IgE internalization facilitated by the formation of IgG/FcγRIIb immune complex [74]. In fact, our study provides evidence that a MEM49- or MED171-based treatment may bring forth this beneficial effect, because we found that both hypoallergens were able to induce strong Met e 1-specific IgG_2a_ responses even a pro-Th2 adjuvant was used during immunization. Such production of specific IgG_2a_ and absence of Met e 1-specific IgE might correspond to the Th1-driving potential of the two hypoallergens. Most importantly, these antibodies were able to significantly block IgE of both shrimp allergy subjects and Met e 1-sensitized mice from binding to Met e 1. Such inhibitory and Th1-inducing potential are beneficial and it is likely that a MEM49- or MED171-based vaccine will modulate shrimp tropomyosin-induced allergic responses.

To our knowledge, this is the first study providing experimental evidence of a shellfish allergen-specific IgG blocking antibodies induced by hypoallergens. Our results demonstrate significant decrease in the *in vivo* and *in vitro* IgE reactivity and allergenicity of the two designer shrimp tropomyosin hypoallergens MEM49 and MED171 when compared to the wild type allergen Met e 1 and more importantly, robust IgG antibodies’ responses with inhibitory potential to Met e 1-specific IgE antibodies of shrimp allergy subjects and Met e 1-sensitized mice. Finally, this work signifies an important discovery that could potentiate the development of prophylactic and/or therapeutic therapies in shellfish allergy.

## Supporting Information

Figure S1 Comparison of the tropomyosin sequences for the construction of hypoallergen MEM49. Tropomyosin sequence of Met e 1 was compared to that of four fish species *Salmo salar* (Atlantic salmon), *Epinephelus coioides* (orange-spotted grouper), *Siniperca chuatsi* (Mandarin fish) and *Thunnus thynns* (Atlantic bluefin tuna). Amino acid deviations within each IgE-binding epitope (framed) were identified and subsequently mutated into the homologous sequence of fish tropomyosins (bold letters shaded in gray) for the construction of hypoallergen MEM49.
(TIF)

Figure S2 Computational prediction of tropomyosin IgE-binding epitopes. **(A)** Surface probability score of each amino acid residue of Met e 1 in Emini Surface Accessbility Prediction. **(B)** Antigenic propensity score of each amino acid residue of Met e 1 in Kolaskar & Tongaonkar Antigenicity. **(C)** Epitope score of each amino acid residue of Met e 1 in Bepipred Linear Epitope Prediction.
(TIF)

Table S1 Clinical characteristics and shrimp tropomyosin-specific IgE of the shrimp allergy patients included in this study. 12 patients 3–17 years old with documented history of shrimp allergy were recruited in this study for mapping the major IgE-binding epitopes of Met e 1 and characterizing the IgE reactivity of the hypoallergens.
(DOCX)
