# Supplementary figures and images for: Immunization with Hypoallergens of Shrimp Allergen Tropomyosin Inhibits Shrimp Tropomyosin Specific IgE Reactivity
Source: PLoS One. 2014 Nov 3;9(11):e111649. doi: 10.1371/journal.pone.0111649 (PMC4218792; doi:10.1371/journal.pone.0111649)

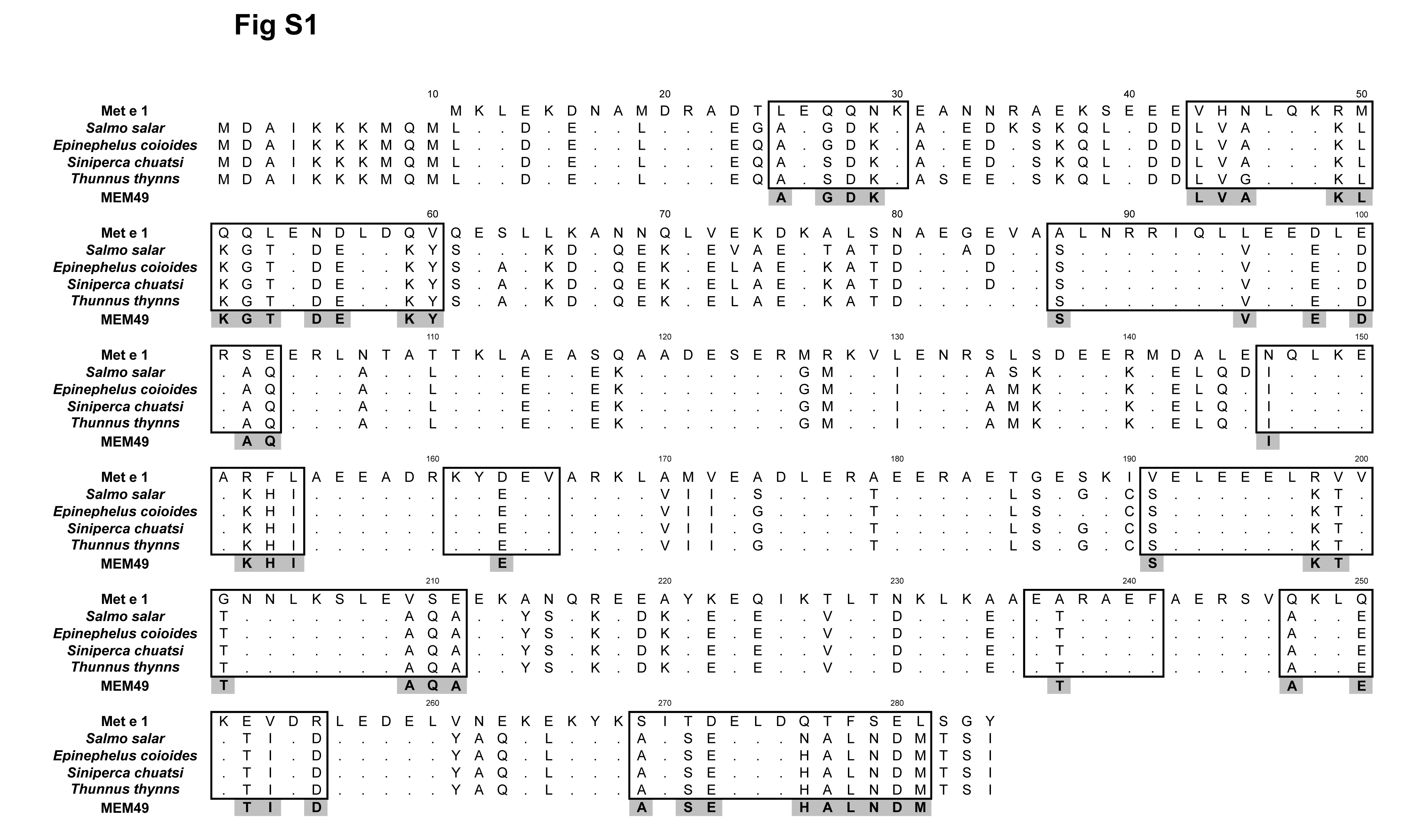

Supplement: Figure S1 — Comparison of the tropomyosin sequences for the construction of hypoallergen MEM49. Tropomyosin sequence of Met e 1 was compared to that of four fish species Salmo salar (Atlantic salmon), Epinephelus coioides (orange-spotted grouper), Siniperca chuatsi (Mandarin fish) and Thunnus thynns (Atlantic bluefin tuna). Amino acid deviations within each IgE-binding epitope (framed) were identified and subsequently mutated into the homologous sequence of fish tropomyosins (bold letters shaded in gray) for the construction of hypoallergen MEM49. (TIF) [file pone.0111649.s001.tif]

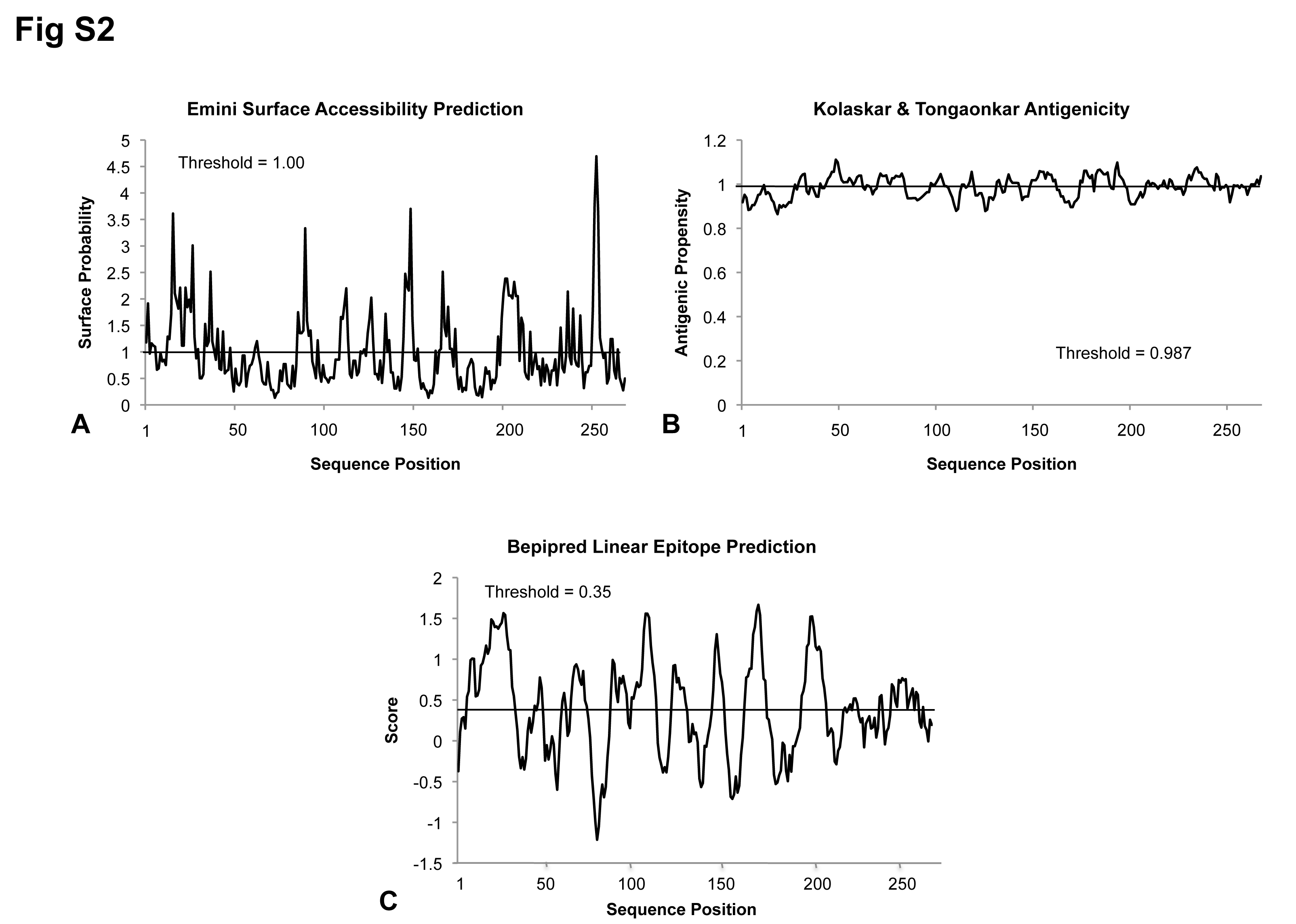

Supplement: Figure S2 — Computational prediction of tropomyosin IgE-binding epitopes. (A) Surface probability score of each amino acid residue of Met e 1 in Emini Surface Accessbility Prediction. (B) Antigenic propensity score of each amino acid residue of Met e 1 in Kolaskar & Tongaonkar Antigenicity. (C) Epitope score of each amino acid residue of Met e 1 in Bepipred Linear Epitope Prediction. (TIF) [file pone.0111649.s002.tif]
