# Supplementary material for: Immunization with Hypoallergens of Shrimp Allergen Tropomyosin Inhibits Shrimp Tropomyosin Specific IgE Reactivity
Source: PLoS One. 2014 Nov 3;9(11):e111649. doi: 10.1371/journal.pone.0111649 (PMC4218792; doi:10.1371/journal.pone.0111649)
Supplement: Table S1 — Clinical characteristics and shrimp tropomyosin-specific IgE of the shrimp allergy patients included in this study. 12 patients 3–17 years old with documented history of shrimp allergy were recruited in this study for mapping the major IgE-binding epitopes of Met e 1 and characterizing the IgE reactivity of the hypoallergens. (DOCX) [file pone.0111649.s003.docx]

**Supporting Information**

**Table S1** Clinical characteristics and shrimp tropomyosin-specific IgE of the shrimp allergy patients included in this study. 12 patients 3- 17 years old with documented history of shrimp allergy were recruited in this study for mapping the major IgE-binding epitopes of Met e 1 and characterizing the IgE reactivity of the hypoallergens.

| **Patients** | **Sex** | **Age** | **SPT (mm)** | **Reaction to shrimp** | **Pen a 1-specific IgE (ImmunoCAP, kU_A_/L)** | **Met e 1-specific IgE (ELISA, 1:5 dilution, OD450nm)** |
| --- | --- | --- | --- | --- | --- | --- |
| 1 | M | 13 | ND | U | 2.04 | 1.22 |
| 2 | M | 8 | 11 | U | 26 | 2.15 |
| 3 | M | 3 | ND | U | 0.97 | 1.37 |
| 4 | M | 5 | 3 | AE | NA | 1.31 |
| 5 | M | 11 | ND | AE | 22.4 | 1.57 |
| 6 | M | 17 | ND | A | NA | 1.24 |
| 7 | F | 11 | 8 | A, AE | 24.2 | 1.69 |
| 8 | M | 15 | ND | AE | 8.44 | 1.21 |
| 9 | F | 12 | 4 | ANA | NA | 1.15 |
| 10 | F | 14 | 10 | ANA | 33.8 | 2.32 |
| 11 | M | 4 | 15 | ANA | 2.97 | 1.01 |
| 12 | F | 5 | ND | ANA | NA | 1.44 |

*SPT, skin prick test
ND*, not done; *NA*, not available

*A*, asthma; *AE*, angioedema; *ANA*, anaphylaxis; *U*, urticaria
